# Understanding STEM academics’ responses and resilience to educational reform of academic roles in higher education

**DOI:** 10.1186/s40594-022-00327-1

**Published:** 2022-01-28

**Authors:** Pauline Mary Ross, E. Scanes, P. Poronnik, H. Coates, W. Locke

**Affiliations:** 1 Melbourne Centre for the Study of Higher Education (Melbourne–CSHE), University of Melbourne, Melbourne, VIC Australia; 2 The University of Sydney of School Life and Environmental Science, Sydney, NSW Australia; 3 Climate Change Cluster, University of Technology, Sydney, NSW Australia; 4 The University of Sydney, School of Medical Sciences, Sydney, NSW Australia; 5 Institute of Education, Tsinghua University, Haidian District, Beijing, China

**Keywords:** STEM education reform, STEM teacher, Teacher resilience, Higher education, Bronfenbrenner’s socio-ecological model

## Abstract

**Background:**

Across the globe, there have been significant reforms to improve STEM education at all levels. A significant part of this has been teacher reform. While the responses and resilience of STEM teachers to educational reforms in secondary education have received significant attention, the responses and resilience of STEM teachers in higher education remains understudied. In higher education, educational reforms of academic roles have seen increasing numbers of STEM academics focussed on education. Responses of STEM academics to education reform of the academic role have some parallels with teacher resilience, but there are also potential misalignments within a culture which values and prioritises science disciplinary research. This study examined the responses of STEM academics in higher education to educational reform of the academic role using the theoretical construct of resilience and Bronfenbrenner’s socio-ecological model. This was a 2-year case study of 32 academics and senior educational leaders in higher education in STEM. Data collection included semi-structured interviews which were theme coded and inductively analysed.

**Results:**

The responses and resilience of STEM academics focussed on education appeared to be dependent on interactions between individual disposition in the microsystem and influences of the exosystem and the external macrosystem. Five major themes emerged about the value and quality, scholarship and expertise, progress and mobility, status and identity and community and culture of STEM academics focussed on education. The exosystem was a significant unidirectional influence on STEM academics where judgements were made concerning academic performance, awards, and promotion. Responses of senior leaders in the exosystem were influenced by the macrosystem and culture of science. Academics focussed on research, rather than education were more valued and more likely to be both financially rewarded and promoted.

**Conclusion:**

During this pressured decade, where COVID-19 has intensified stress, more attention on the direction and reciprocal relationships in the socio-ecological model of higher education is needed in order for educational reform in higher education STEM to be effective. Resilience of STEM academics to educational reform in higher education is a dynamic quality, and the capacity to “bounce back”, learn from challenges, and realise expectations of educational reform will depend on an understanding of resilience and support of Bronfenbrenner’s spheres of influence.

## Introduction

Across the globe, there have been significant reforms to improve STEM education at all levels. Over a decade ago, leading science and nature journals along with Nobel Prize winners called for profound change, and a departure from the well-established STEM culture in higher education which rewards research over teaching (Anderson et al., 2011; Savkar & Lokere, 2010; Schmidt, 2012, 2019; Wieman, 2007; Wieman et al., 2010). In response, teacher reform initiatives in higher education led to the establishment of teaching pathways for STEM academics. Depending on the country, these teaching pathways and academics are known by different titles. In the United States of America (USA), these academics are known as Science Faculty with Education Specialties (SFES) (Bush et al., 2008); in Canada, they are known as Teaching Stream Focussed (TSF) (Vajoczki et al., 2011); in the United Kingdom (UK), the titles differ depending on level, and include Teaching and Senior Fellow (Locke, 2014); and in Australia, these academics are known as Teaching or Education Focussed (Probert, 2013, 2015). These teaching pathways have increased across the higher education ecosystem at a rapid rate (Jung et al., 2014; Locke et al., 2016; Marini et al., 2019; Teichler et al., 2013; Whitchurch, 2019; Whitchurch & Gordon, 2010, 2013), and already, in the USA and the UK, less than 50% of academics remain in academic roles which include disciplinary research (Locke, 2012, 2014; Teichler et al., 2013), compared to 59% in Australia (Bexley et al., 2011; Probert, 2013, 2015; Ross, 2019). In the last decade in Australia, academics in teaching pathways have increased by 300% (DET, 2017, various years).

Educational reforms to the academic role sit against the history of the modern university, which since inception has combined disciplinary research and teaching, where one without the other was considered unconscionable (Barnett, 1992a, 1992b; Brew, 1999; Brew & Boud, 1995; Feldman, 1987a, 1987b; Hattie & Marsh, 1996, 2004; Marsh, 1980, 1987; Marsh & Hattie, 2002; Naidoo, 2005; Neumann, 1992; Ramsden & Moses, 1992; Robertson, 2007; Scott, 2004, 2005; Teichler et al., 2013; Trigwell, 2005; Utll et al., 2017). However, there remains a lack of evidence that success in one is beneficial to the other (Hattie & Marsh, 1996). Hattie and Marsh (1996) and Marsh and Hattie (2002) found no relationship between research and teaching quality, they argued against “teaching-only” pathways because whereas time on research improves research quality, time on teaching does not necessarily improve teaching quality (Hattie & Marsh, 1996). Notwithstanding, with professional development and feedback, teaching quality can be improved (Gibbs, 2010; Gibbs & Coffey, 2004).

More contemporary commentators continued to emphasise that students benefit from research when they are active participants (Healey, 2005), active researchers are more likely to include research-related practices in their teaching (Magi & Beerkens, 2016) and academics pursuits between teaching and research are associate with work stress and beliefs (Daumiller & Dresel, 2020). The necessity of research in an academic role for teaching quality has not been unequivocally demonstrated. In fact studies provide evidence that academics who excel in research do not make better teachers (Bak & Kim, 2015; Gibbs, 2010; Norton et al., 2013), departments that excel in research do not have better student experience (Norton et al., 2013; Ramsden & Moses, 1992), and research-intensive institutions do not have greater student satisfaction (Gibbs, 2010; Jensen, 2014). Moreover, others have commented that it is not possible for one person to excel in teaching and research (Bexley et al., 2011; Friedrich & Michalak, 1983; Gamble, 1985), and although anecdotal evidence suggests the contrary (Schmidt, 2019), the argument that research and teaching are incompatible and should separate has gained traction (Barnett, 1992a, 1992b). It therefore seems questionable that STEM academics should continue to require research performance as a prior condition for good teaching; indeed, even arguments that research is a necessary prerequisite for a STEM academic to be ‘across’ new developments in the field, so as to stimulate thinking in students, have failed to gain traction (Boyer, 1990; Coates & Bexley, 2016; Friedrich & Michalak, 1983; James et al., 2013; Whitchurch, 2019).

Nevertheless, one cannot ignore that the removal of disciplinary research from the academic role in STEM comes with consternation. There is evidence that the cornerstone of identity for STEM academics remains disciplinary research. Academic identity is constructed and consolidated in discipline based undergraduate, doctoral, and post-doctoral studies (Henkel, 2005, 2007). Success in STEM research is a driver of prestige and status, and also builds reputations (Anderson et al., 2011; Mervis, 2013; Savkar & Lokere, 2010; Schmidt, 2012; Wieman, 2007; Wieman et al., 2010). The primacy of research can be observed at scientific professional conferences, where education sessions suffer from low attendance—being scheduled at times which are in conflict with the main part of a given conference—thus reinforcing the perception that education is both not valued and also essentially separate to the main purpose of conferences (Brownwell & Tanner, 2012; Mervis, 2013).

The danger is that without disciplinary research in a STEM academic role, education-focussed STEM academics may have less autonomy, experience more restrictions, be lower in status, and have less overall value, even though, paradoxically, they do the work which brings in teaching income and ultimately pays the majority of staff salaries. As a consequence, the academic workforce may polarise into research STEM academics who receive higher status and greater autonomy, and are ultimately more valued than education-focussed STEM academics who do the bulk of the teaching and administration (Henkel, 2005). Such a dynamic could impact women more than men, the former of whom are already known to take on a greater share of teaching workload and administration (Thomas & Davies, 2002; Trowler, 1998) and are consequently at risk of ending up as second-rate citizens because of the lower status of teaching (Forster, 2001; Thomas & Davies, 2002). The perception that the pastoral care side of teaching comes “naturally” to women, rather like “domestic work”, only exacerbates this dilemma, likely occasioning the further entrenchment of the existing underrepresentation of women at senior levels in academia, especially in STEM (Bell, 2009, 2010; Diezmann & Grieshaber, 2019; Ross, 2021).

Boyer (1990) stated that the solution to this is the broadening of the academic role, so as to encompass the Scholarship of Teaching and Learning (SoTL) that evaluates the effectiveness of teaching and learning initiatives. Further Boyer (1990) argued that it is not necessary for all aspects of academic work to be done by each academic, rather some academics can research and evaluate the effectiveness of their teaching. Others such as Barnett (2011) agree and go further, emphasising that as long as someone is doing the research in or outside the university, teaching can be research-led. As a result, SoTL has become an international movement, along with Disciplinary Based Education Research (DBER). DBER faculty are defined as academics who investigate learning in a discipline and generate insights into students’ learning (NRC, 2012).

Responses of academics to this educational reform of the academic role in higher education have been both positive and negative. Negative responses include academics in education-focussed roles being anxious about workloads, about how others perceive them, and that wrong identity might be imposed (Brownwell & Tanner, 2012; Chalmers, 2011; Flecknoe et al., 2017). Positive responses include optimism about their new role as a rebellion against the conventional, which will bring opportunity and perhaps some greater flexibility to explore education research (Flecknoe et al., 2017; Probert, 2015). Flecknoe et. al. (2017), in a survey of nine newly appointed education-focussed academics at one research-intensive university, found that the primary motivation of academics was to follow a career path they were interested in and were good at, while also highlighting the perceptions of the roles by traditional academics and their lack of skills and limited experience in educational research. Studies have similarly reported academics as being more likely to have imposter syndrome, feeling they are now in a field where they do not belong (Clance & Imes, 1978). Education-focussed roles require a unique set of skills and knowledge, which at first can be difficult and confronting to learn (Simmons et al., 2013). STEM academics may find traversing the boundary between disciplinary research and SoTL or education research most difficult, because their prior training has involved distinct modes of inquiry with a high degree of quantitative approaches and general agreement on the methods of hypothesis testing and data collection analysis. This may not be the case for academics in the social sciences, where scholars tend to commonly disagree and the process is one of argumentation (Gardner & Willey, 2016; Jones, 2011). Hardre et. al. (2011) note that when an academic joins a new community, they do not immediately take on its values, but rather bring their own “baggage” from previous communities. Academics that transition into education-focused positions often describe a moment of clarity, or an “identity threshold”, where they suddenly begin to see the bigger picture and feel that they can contribute to teaching and learning (Simmons et al., 2013). The longer education-focussed academics are in these roles, and as the distance between the creation of new knowledge and the transmission of knowledge widens and connections to disciplines fade, academic identity may become chimeric (Bennett et al., 2016). STEM academics may struggle in education-focussed roles because their field of research is strongly linked to their science identity.

If STEM education reform is to be successful and deliver on expectations or improved student experience, we need to know more about the responses to challenges that STEM academics face in education-focussed pathways within higher education. Academics, regardless of their role, experience adversity—even the most successful. Adversity is ubiquitous in a range of academic activities, including through the rejection of research grants and academic publications (Day, 2011; Lee et al., 2021). Lee et. al. (2021) provide at least four relevant challenges for academics: (i) balancing an academic workload; (ii) casualisation of the workforce; (iii) the managerialism phenomenon, and (iv) transition from field of practice to academia. Chan et. al. (2020) summarise the feelings of many academics: “It [the job] is very stressful, it’s all encompassing, it never stops, it’s relentless, it’s full of rejection, it’s full of stress, it’s full of criticism”. Chan et. al. (2020) describe those academics who respond positively to rejection with thoughts like “I can learn from this” rather than “I’m useless” as more likely to be successful. Academics in education-focussed pathways will also experience the challenges of student evaluations and their bias (Fan et al., 2019; Kaschak, 1978) and new research paradigms and to survive will need resilience.

Research on the resilience of teachers in secondary education emerged to better understand how to increase retention of motivated and talented teachers (Gu, 2014; Gu & Day, 2007, 2013; Luthar & Brown, 2007; Ungar, 2012). Gu and Day (2007) described three reasons why resilience is important for teachers. First teachers are role models for their students and cannot expect their students to be resilient, if they themselves are not. Second, teachers need to sustain the motivation to teach, often large numbers of students over several years. Third there is a tight link between teacher resilience and student performance. Studies identified the significance of the relational aspect of teacher resilience, which depends on trust and support, being present in the social and organisational structure of the school, and further identified the flow on effects of this for the performance of students (Beltman et al., 2011; Gu, 2014; Mansfield et al., 2016; Ungar et al., 2013). Although, originally, the individual aspect of resilience was emphasised, current understanding is that teacher resilience is relational and a multidimensional construct, where the personal resources of an individual teacher—such as self-efficacy and self-esteem—interact with the professional context and external environment (Beltman et al., 2011). When these interactions are positive, they result in teacher well-being and job satisfaction. When they are less than positive, they result in teacher burnout (Beltman et al., 2011).

While responses of STEM teachers to educational reforms have received significant attention at secondary education, responses of STEM academics to education reforms in higher education, particularly responses and resilience of STEM academics in education-focussed roles, remain understudied. Resilience of STEM academics in education-focussed roles will be critical if educational reforms are to be successful. While research on teacher resilience has some alignment with academics in higher education, there are also some aspects of misalignment. Perhaps foremost, academics interest and raison d’être, is to do disciplinary research, rather than teach (Teichler et al., 2013). Further, in contrast to teachers who work towards collective institutional goals, academics are highly individualistic in increasingly competitive contexts. When rewards are given, such as promotion, they are provided because academics have achieved individual goals generally in research, which add to institutional reputation and performance. Academics are also less reliant on the trust and support provided internally by the social and organisational structure of the university. Furthermore, academic perspectives and values are frequently mismatched with institutional stratagems and corporate visions (Winter, 2009; Winter & O’Donohue, 2012). Academics draw on resources more often from outside rather than inside the university, with those colleagues inside the university often being their direct competitors.

## Theoretical underpinnings

### Resilience

Resilience is acknowledged to be a multidimensional—multideterminant, complex construct, which can be approached at multiple levels of analysis (Southwick et al., 2014). Broadly defined, resilience is the capacity to “bounce back” and recover from adversity or stress (Carpenter et al., 2001; Folke et al., 2004; Frydenberg, 2014, 2017; Gunderson, 2000; Karlson, 2013; Masten, 2001; Southwick et al., 2014; Walker, 2019). Walker (2019), and others emphasise that resilience is not just about recovery or “bouncing back” but, importantly, also includes the capacity of an ecosystem or individual to “learn from” or “adapt” to stress (Walker, 2019, 2020). Resilience is also the capacity to endure, overcome, and learn from repeated or cumulative stress, rather than from a single adverse event (Carver, 1998; Earvollino‐Ramirez, 2007; Masten, 2001; Schoon, 2006; Tugade et al., 2004).

**Fig. 1:**
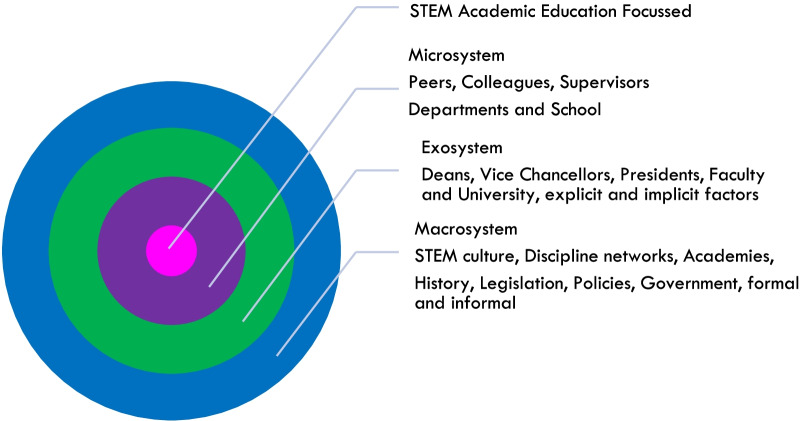
Bronfenbrenner’s socio-ecological model representing the layers of influence on STEM academics focussed on education in higher education

Bronfenbrenner (1977) situated human development and resilience in a social or bio-ecological framework, extending beyond reliance as an individual construct. Bronfenbrenner (1977) suggests that the resilience of an individual depends upon a nested set of relationships, shown as a series of concentric circles; the microsystem, the mesosystem, the exosystem, the macrosystem, and the chronosystem. At the centre of the circle is the individual, and other circles then span out from the individual and represent the levels of influence contributing to individual resilience at each level of the five-level system (Bronfenbrenner, 1977; Bronfenbrenner & Ceci 1994; Guy-Evans, 2020). The first circle is the microsystem, where the interactions between the individual and the environment are bi-directional; each influence and can change the opinion of the other. The mesosystem is the interactions among microsystems, i.e. in a higher education context the interactions between academics and their peers. The exosystem is the environment which does not directly contain the individual, but has significant influence, i.e. decisions made by heads of school and deans. The macrosystem is the influential culture in which an individual is immersed, which influences belief, i.e. the culture of STEM. Lastly the chronosystem and events which influence individuals and occur over a lifetime, i.e. a life changing event of COVID-19 or changing workplaces (Guy-Evans, 2020; Figure 1).

Bronfenbrenner’s work shifted views of resilience from a characteristic of an individual to the interactions of the personal characteristics of an individual and the factors that facilitate resilience. Ungar (2012), drawing on Bronfenbrenner, proposes that resilience of an individual is a reciprocal interaction between the quality of the environment and the individual; the individual is influenced by the environment and, in turn, the environment influences the individual (Frydenberg, 2017; Ungar, 2012). Importantly, as Ungar et. al. (2013) state, “this way of conceptualising resilience means that individuals are not always the most important locus for change in a complex system” (p. 357). Resilience instead may have far more to do with the environment than at the level of an individual (Ungar et al., 2013).

Overall, there is a need for greater understanding of the responses of STEM academics to educational reform of academic roles. The theoretical construct of resilience and the Bronfenbrenner socio-ecological resilience framework was used to view and understand the influences and experiences of education-focussed STEM academics across all career stages (Fig. 1). Understanding the influences and interactions between the micro, exo and macrosystems will help to build resilience of STEM academics and improve the conditions for careers based in education-focussed academic roles.

This study contributes to the limited theoretical understanding of the development of STEM academics who have trained in disciplinary STEM research, yet are no longer active in disciplinary STEM research, and now need to navigate in an education-focussed role the development of a new epistemology and identity.

## Methods

The aim of this study was to examine the responses of STEM academics in higher education on education-focussed pathways, and senior leaders responsible for creating education-focussed pathways, using the theoretical construct of resilience.

A series of semi-structured interviews were held with 32 academics—tenured faculty members at a range of levels and senior leadership roles in education at Australian universities. This included 11 males and 21 females (34:66 male-to-female ratio). The greater proportion of females perhaps reflecting the gender-skewed ratio among education-focused STEM academics (Ross, 2019).

Academics interviewed were in a variety of roles and levels, from teaching and research roles to academics who had transitioned from teaching and research to education-focussed (education focussed) roles or teaching and research academics whose research area was disciplinary-based education research (DBER). Overall, there were 11 research and teaching focused academics (both disciplinary and disciplinary-based education research) and nine education-focused academics (with education-focussed research expectations). Academics interviewed were representative of Science Technology, Engineering, Mathematics and Medical Science (STEMM) disciplines, specifically Biology, Chemistry, Mathematics, Medical Sciences, Physics and Psychology. Academics from the disciplines of Biology, Mathematics, Medical Science and Physics were well represented. Academics in senior leadership roles were also interviewed for their perspectives on education-focussed roles, and these included a Dean of Science, Pro Vice Chancellors (Education), Deputy Vice Chancellor (Education) and a Vice Chancellor and Principal and represented a range of STEMM disciplines. The range of academic roles and levels of academics interviewed are detailed in Table 1. These academics ranged in levels from mid-career faculty members to experienced faculty members and included: one lecturer (level B), four senior lecturers (level C), 12 associate professors (level D) and 13 full professors (level E) from 10 universities in total with three from Innovative Research Universities (IRU) and Australian Technology Network (ATN) universities with six from research-focused Australian universities or Group of Eight (Go8) (representing 75% of all research-focussed institutions), including the two research-focused universities where education-focused academic roles were first introduced in 2006 and 2009, respectively. Senior leaders were equally distributed across research and non-research-focussed universities and disciplines. These academics were readily identifiable using established discipline networks across the Science disciplines. Research Ethics clearance was applied for using the Australian National Ethics Application Form (NEAF) process (now replaced by the Human Research Ethics Application HREA) and assessed by Human Research Ethics Committee at Western Sydney University, approval number H11177.

**Table 1:** Number and identification of interviewees from universities classified as research intensive (Group of Eight, Go8), Australian Technology Network (ATN) or Innovative Research University (IRU)

Interviewee number	Name of institution	Classification of university	Level of academic	Gender
1	Australian National University	Go8	E	M
2	Monash University	Go8	D	F
3	University of Tasmania	N/A	C	F
4	Office of the Chief Scientist	N/A	N/A	F
5	University of Melbourne	Go8	D	F
6	Western Sydney University	IRU	E	M
7	University of Technology, Sydney	ATN	E	M
8	University of Queensland	Go8	D	F
9	University of Sydney	Go8	E	F
10	University of Queensland	Go8	D	F
11	Australian National University	Go8	E	M
12	University of New South Wales	Go8	D	F
13	University of Queensland	Go8	C	F
14	Australian National University	Go8	E	F
15	Australian National University	Go8	E	M
16	Flinders University	IRU	E	F
17	Australian National University	Go8	D	F
18	Australian National University	Go8	E	F
19	University of Queensland	Go8	D	F
20	Monash University	Go8	E	F
21	Monash University	Go8	D	M
22	University of Queensland	Go8	B	F
23	University of Queensland	Go8	D	F
24	University of Sydney	Go8	E	F
25	University of Queensland	Go8	C	M
26	University of Technology Sydney	ATN	C	F
27	Monash University	Go8	D	M
28	University of Sydney	Go8	E	M
29	University of New South Wales	Go8	D	M
30	Private Organisation	N/A	N/A	F
31	University of Technology, Sydney	ATN	E	M
32	University of Sydney	Go8	D	F

Academic level of appointment (from Professor level E to B mid-career academic) and gender (male, female) is provided

N/A not applicable

**Fig. 2:**
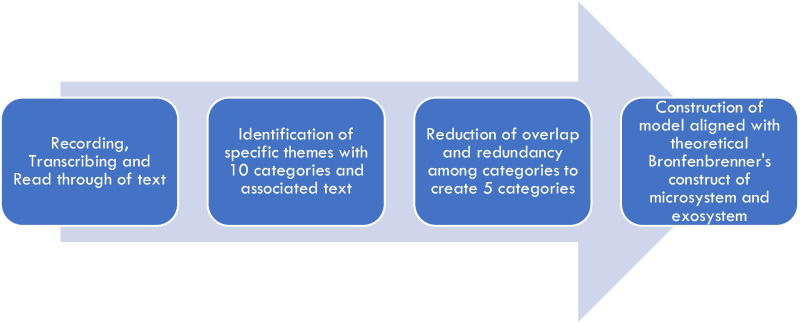
Inductive analyses used to code transcribed themes, following Thomas (2006, p. 6)

A series of 15 questions (Table 2) were used to create a scaffold in semi-structured interviews, lasting between 60 and 90 min, and which were recorded. Following recording, the interviews were transcribed by a professionally accredited transcription service (Pacific Transcription Co. Milton, QLD, Australia). Inductive coding was done to identify themes through careful reading of the text by two independent researchers using the approach of Thomas (2006). Text was identified and categorised into ten emergent themes. Overlapping themes were considered and redundancies merged to create five final themes (Fig. 2). The five themes were (1) value and quality, (2) scholarship and reputation, (3) progress and mobility (4) status and identity and (5) community and culture (Table 3). Following this, these themes were aligned to Bronfenbrenner’s framework and the findings reported at the microsystem and exosystem levels (Fig. 1).

**Table 2:** Questions asked to academics in semi-structured interviews

Question number	Question
1	With reference to your own university, to what extent is there a strong emphasis on disciplinary research and publication?
2	With reference to your own university, to what extent is there a strong emphasis on learning and teaching and publication?
3	With reference to your own university describe the extent to which academics focused on learning and teaching are valued? What are the indicators of this?
4	With reference to your own university describe the extent to which the quality of teaching is valued? What metrics are used to provide evidence of value and quality?
5	With reference to your own university, describe the extent to which disciplinary science research is valued? What metrics are used to provide evidence of value and quality?
6	With reference to your academic role, how much time in your role did you spend this semester on teaching, administration and disciplinary science research/scholarship?
7	Describe some of the metrics which should be used to evaluate education-focused positions and/or education component of the academic role in science
8	Should universities appoint academics in education-focused positions, separately to disciplinary research? Explain reasoning for your answer
9	Flexibility in the academic role in the sciences is being broadly discussed? To what extent is flexibility possible in the academic role? For example is it possible to move from focus on research, to education and back again? What metrics and/or evidence is used to provide evidence of value and quality?
10	Which source of metrics and/or evidence (i.e. student evaluation, peer evaluation, awards, student progression) do you most value when evaluating the quality of your teaching?
11	What is meant by the scholarship in teaching and learning?
12	Are you published in learning and teaching? If so what value does it add to your role as an academic?
13	What factors are primary in driving your decisions to work in education, research or both? Considering your academic work, how many hours to you spend in a typical week on the following activities (a) Teaching (b) Research (c) Service (unpaid assistance to government agencies or colleagues) (d) Administration (unit co-ordination, committee meetings) (e) Other (attending conferences, reviews)
14	Considering your academic work, how many hours to you spend in a typical year on the following activities (f) Teaching (g) Research (h) Service (unpaid assistance to government agencies or colleagues) (i) Administration (unit co-ordination, committee meetings) (j) Other (attending conferences, reviews)
15	Considering promotion and promotion committees which of the following activities is likely to influence the decision (a) Teaching (b) Research (c) Service (unpaid assistance to government agencies or colleagues) (d) Administration (unit co-ordination, committee meetings) (e) Other (attending conferences, reviews)

**Table 3:** Identification of themes, reduction of overlap and redundancies and alignment of themes with Bronfenbrenner’s model

Number	Coding 1	Coding 2	Overlap	Final themes
1	Mixed messages	Value	Value, expertise and merit	Value and quality
2	Expertise	Expertise		Scholarship and expertise
3	Reputation	Reputation	Reputation, scholarship, research and funding	
4	Flexibility and mobility	Scholarship		
5	Funding	Funding		
6	Progress and promotion	Progress and promotion	Progress, promotion, merit and metrics	Progress and mobility
7	Education research	Education research		Status and identity
8	Community	Community	Community	Community and culture
9	Students	Students		
10	Merit	Metrics and merit		

## Results

### Value and quality

STEM academics in education-focussed roles described views that the rhetoric around the value of their role did not match reality and instead actions of senior leaders reflected the real and lesser value of education-focussed STEM academics compared to STEM research academics. The official view of senior leaders was that teaching is valued, but the “real” view suggests that it is less valued than overtly expressed.

“I can give you a view of what we officially, you know, that we think teaching and research are equally important.” Interviewee 1, Level E, Go8

“I think if the rhetoric that we hear was mirrored accurately by the things that happen then we would be on the right track.” Interviewee 7, Level E, ATN

STEM academics expressed views that teaching workload can be overwhelming and the only activity that they were able and should do.

“a reasonable workload is quite difficult to manage.” Interviewee 8, Level D, Go8

**“**So we’ve got these teaching people and I want to do my research so we’ll just give more of the teaching to them. What else would they be doing anyway?” Interviewee 5, Level D, Go8

They also were aware that STEM academics in education-focussed roles were historically a place to locate academics underperforming in research and if the potential problematic dual system if STEM education-focussed academics were regarded as second-class academics. They described the unlikelihood that STEM academics in education-focussed roles would ever be viewed as equivalent or as valued as research academics, especially at research-intensive universities, but the ideal where both would be equally valued.

“It shouldn’t be a place where you shunt people who aren’t performing as you would wish elsewhere, and it shouldn’t be seen as a place where you dump a whole heap of first year teaching or something because not one else wants to do it. Also I think if you’re going to have those position they have to have equivalent status. That’s probably never going to happen.” …. Interviewee 14, Level E, ANU

“I think if you do that then you start to create a bit of a ghetto don’t you. I think that’s what people are really scared about is that you go into this institution of the future where you have a whole bunch of people who just do the teaching and a whole bunch of people who just do the research because we can see how it would go that way.” Interviewee 5, Level D, Go8

“We should change. We should change and we should actually appoint across all levels with speciality in education.” “That’s been a disaster….. they are failed researchers who are actually kept on. In fact they are often valued more than people who make effort and do brilliant educational innovation in teaching and learning.” Interviewee 32, Level D, Go8

STEM academics in education-focussed roles, also expressed doubt about whether academic leaders could judge education quality. For example, there was scepticism about the use of student evaluations to measure quality in education-focussed roles and capacity of promotion panels to judge effectiveness. This was in clear contrast to indicators of expertise in disciplinary research which can be readily identified from a deep understanding of the discipline and quality measured by the creation of new knowledge and patents and publications which is supported by grant success, publications and the H-Index. Expertise in education-focussed roles was not readily understood and this was especially obvious during the promotion processes.

“I applied for a promotion unsuccessfully at that level was, you need to have more journal publications. I also was told that, we would expect that your teaching evaluations would be higher, which is not something that is in the criteria that are written down. It just seemed an expectation that student evaluations will be better than some imagined level while you’re doing a heavy teaching load, and you’re establishing, and becoming a national figure in education research, or Scholarship of Teaching and Learning in your disciplinary area.” Interviewee 22, Level B, Go8

“I think it’s very hard to demonstrate learning in a robust, scientific way. How—what I did just caused someone else to have great learning. That’s, I think, a very tricky thing to make that very clear cause and effect link to. But I do actually—I am increasingly worried when we start to move very individualistically to look at me, I’m a good teacher. I need to prove that to you and now I need to prove that to the promotion committee—I am a good teacher.” Interviewee 10, Level D, Go8

“Are students the best judge of whether they’ve had a good education experience? Students’ evaluations are what we use here and there’s lots of problems with that. We all look at the students’ scores with a somewhat sceptical eye.” “She’s a great lecturer so she’ll get good students’ scores but she also has this immense body of knowledge and she influence so many other people and how do we quality that? I’m not sure we do. How do you put metrics on the sort of—that sort of impact”. Interviewee 11, Level E, Go8

STEM academics were also aware of central funding processes available to head hunt and recruit high-performing researchers by appointment, while this rarely happened for high-performing education-focussed STEM academics.

“A high profile researcher and his team will be ‘parachuted’ into the School (i.e. they are being poached from elsewhere), these sorts of parachuting appointments prevent filling desperate holes in teaching but will increase total research performance….. they are typically for research only appointments, and are never done for high profile teachers”. “What is more insidious is those people that there is no advert for. They actually appear because they have been head-hunted and it’s all done very secretively. Done behind closed doors. There is no general knowledge to why the person is employed what the criteria were”. “No-one [who] comes through the door by stealth is a high quality teaching and learning expert in science. That never ever happens”. Interviewee 7, Level E, ATN

Not only was there an absence of education-focussed academic appointments, there was also evidence provided that STEM education-focussed academics would be appointed at lower academic levels and rarely at the level of a tenured professor.

Senior leaders were aware of the challenges for STEM academics in education-focussed roles.

“There’s a gap here is what I’m saying. I think between the perception of how highly valued a teaching or education-focussed academic in the science mathematics disciplines is, compared to a research focussed. I think this is one of the challenges of bridging this gap”. “I value those who decide to focus their effort in education and teaching, and the scholarship related to teaching and do that very, very well. I think they should be highly valued for that commitment”. Interviewee 6, Level E, IRU

### Scholarship and expertise

STEM academics in education-focussed roles described the real challenge to upskill in educational research while over time being de-skilled in disciplinary research.

“…with the current focus on research, there is not the same attention to the teaching and learning function in universities; there is a danger of ‘de-skilling’ education focused academics.” Interviewee 7, Level E, ATN

STEM academics who were more readily able to recognise and bridge the paradigm gap between education and science disciplinary research and develop a new set of skills, progressed and were promoted. Many, however, did not have the capacity, interest or time to bridge the gap and experienced significant challenges in progressing. All experienced struggle and rejection of research outcomes, and isolation and suspicion of lower quality research outputs. Some academics progressed despite the obstacles, while others remained in a liminal state for several years.

“My ethics person came back, this person should do discourse analysis. If I knew more of the methods, I think I would have made stronger and more positive contribution like when I was working in that area. You know, I think my earlier career was a bit floundering not having the tools I needed.” I'm trying to align, it's the only way I can survive because I can't survive in any other capacity and I feel like I'm on one of those racks and I'm being torn, but I think that's a common feeling in a way. I don't know, but that's how I feel. I feel like someone's got my arms at one and my feet at the other end and I remember thinking, what sort of life is this.” Interviewee 22, Level B, Go8

“I stepped into education, and I said okay, now I want to supervise PhDs students, I was to seriously supervise honours students, I want to publish in this area then I said to myself I’ve got to do something about it. So I did a EMED research methods course… we can’ expect someone to come in and do physic research without doing sufficient course work to find out what its’ about. Why should we think we can step into somebody’s else’s domain”? Interviewee 32, Level D, Go8

Senior leaders, expressed uncertainty about the value of education research done by STEM academics in education-focussed roles because education journals suffered from low impact factors, citations and could not be counted in excellence in research assessments. They questioned whether STEM academics in education-focussed roles had the capacity and skills to create education research of sufficient quality that would merit the investment of time.

“A lot of these publications are not very, I don’t know what the word is, not very good in terms of they don’t [get] cited. There’s quite a lot of dross in the education literature. They’re in poor quality journals, but the authors will say but you’ve been encouraging us to do this. You’ve said if we’re education focused we need to be engaging with research publishing literature so we have been doing that.” Interviewee 11, Level E, Go8

“I’ve also heard from people who are in that space doing their education research. It’s one of their complaints about it, that it is regarded as a softer scholarship than the normal science that we’re used to. We recognise these journals may have a low impact factor, but look at the potential for them to have a huge impact on our students on their learning outcomes on their employability and even on our ability to recruit new students.” Interviewee 31, Level E, ATN

Some senior leaders realised the solution was to consider at the level of the university rather than individually.

“For any university in aggregate terms—in overall terms—it is vitally important that we have—and we maintain—a strong research program”. Interviewee 6, Level E, IRU

### Progress and mobility

Responses of academics to failure in promotion was a general feeling of failure which caused initial deep hurt. While some academics paid attention to decisions of promotion committees and waited for years to go for promotion others ignored the rejection of committees. Many academics commented on the double standards of promotion committees and their general lack of understanding of education-focussed roles. The academics who were able to ignore and yet pay attention to the feedback from the promotion committee, went onto play the game and provide the committee with what they expected to see. They also recognised the need to be proactive so that the right decisions were made.

“It has to be a two-way street. There’s one way to build up the teaching focused sort of people, but you can’t still let the T&R people keep getting promoted, when they’re barely falling over the bar on teaching. They wouldn’t get promoted if they were barely falling over the bar on research.” Interviewee 5, Level D, IRU

“How would you— what would you evaluate of yourself to put down to demonstrate that you’ve been effective in your role for promotion?” “Publications, grant funding. Again, this is largely I’m being evaluated by research people who value those things.” Interviewee 13, Level C, Go8

“I think they use metrics that they know and that’s what happened to me, because I wasn’t promoted so they used the metrics that they knew and it did not apply to me. So they in grant of them did not have a matrix that could be applied for me and they asked me all these questions….they said they couldn’t understand what my intellectual contribution was, but they should say I had discovered this blah in my individual original contribution. We are pushing the boundaries and we’ll get there.” Interviewee 32, Level D, Go8

“They (teaching-focused academics) have to be more proactive and they need to go have conversations with people on the promotion panel. I know a lot of people on the promotion panels in science and they always tell people come and talk to us. We want to help you. But most people I don’t think actually take up those offers.” Interviewee 10, Level D, Go8

Senior leaders were committed to and had created pathways to promotion for STEM academics in education-focussed roles.

“I believe it’s possible to make a case to become a full professor with a career that’s been about a strong commitment to teaching.” Interviewee 6, Level E, IRU

“The institution made a good step and provided people with a very scaffolded multilayered step approach to presenting portfolios of achievement.” Interviewee 18, Level E, Go8

### Status and identity

STEM academics were aware that and education-focussed role had a lower status that STEM academics focussed on research and that ultimately to be successful an adaption in academic identity was required away from disciplinary research to educational scholarship and research, but this would come with challenges of capacity and time available.

“So, I think if you’re going to have those positions they have to have equivalent status. That’s probably never going to happen.” Interviewee 14, Level E, Go8

“Capacity, and what I mean by that is time. Two things; time, because unless most of my science colleagues, they’ve got a research lab with layers of people in it, so they’ve got PhD students, undergraduate students, postdocs and/or honours students. My sudden—and this happened at my appraisal this year, my Head of School said, whoa, you’ve suddenly got four publications in the last year, you’ve had zero to one in the prior years, what's the difference? It's honours students, I've had a stream of honours students, one a year for the last four years. So suddenly I have capacity to do research. Instead of me trying to do everything, I have people who can help me to do it, or people who I can coach to do it.” Interviewee 13, Level C,’Go8

It was clear that some senior leaders held the view that science research reputation was the priority and primary role of the university.

“Academic staff member at … has to be a serious researcher—seriously engage with research. There is not really room for teaching only appointments here”….. reputation is very. Very important to us”. “We need to do well in the research assessment exercise if we are going to claim to be the best research university”. “For us reputation is more important than that [money]”. Interviewee 11, Level E, Go8

### Community and culture

STEM academics readily recognised disciplinary researchers already had in-built honours and HDR students, ECRs and MCRs who shared interests and were provided with significant resources and they would need to develop a similar but different community. Others experienced the lack of a critical mass within their own institution and the significant challenge of isolation in their home departments, unable to create strong communities, despite often being located next door to disciplinary researchers.

“They had to change research area…start doing it by yourself, not by joining an established group which is already doing research, which already has a direction, which already has a track record of publishing, which already has a track record of acquiring grants” Interviewee 22, level C, Go8

“We exist in the community. We’ve got post-docs. We’ve got students. We’ve got other people we’re talking internally and externally. There’s a whole community that is formed from what we do, whereas someone who comes into an education focussed role and looks after first year biology, they don’t have students under them. They’re unlikely to have PhD students. They don’t draw on a community of people.” Interviewee 9, Level C, Go8

Many expressed the view that STEM education-focussed roles were dominated by women in a culture which favoured males, the academics with the lowest value were those academics that were women in an education focussed.

“Because he’s a boy and he gets on. Our head of school is—runs the place a bit like a boys’ club, he would be the last person to admit that. He thinks he’s very kind to women and the director of education thinks he’s very kind to women too and he is kind but his view of what women can do is I should help women, not I should provide opportunity for challenges for women.” Interviewee 2, Level D, Go8

Senior leaders were silent on issues around community and their role in supporting a cultural change in perception of STEM education-focussed roles in higher education.

## Discussion

No matter what the role, to be an academic requires resilience. The aim of this study was to examine the responses and resilience of STEM academics to educational reform of the academic role in higher education using the theoretical lens of resilience and Bronfenbrenner’s socio-ecological model. Resilience is broadly defined as the capacity to “bounce back”, and “learn from or adapt” to stress (Carpenter et al., 2001; Frydenberg, 2017; Gunderson, 2000; Walker, 2019, 2020). Walker (2019, 2020) states that we may be born with a biological basis for resilience, but resilience is about learning to adapt in difficult and different adverse situations (Walker, 2019, 2020).

The findings of this study reveal that STEM academics focussed on education face adversity. They face adversity in a higher education system which is uncertain about their value and expertise, unlikely to give them equivalent status to STEM academics in disciplinary research roles and confused when judgements of quality need to be made. Within this system, however, responses of STEM academics in education-focussed roles provide evidence of their resilience and capacity to persist. Where does this resilience come from? Is it a personal capacity from the individual academic to respond and the bi-directional interactions in the microsystem and/or influences of peers and supervisors within schools and departments in the mesosystem? Is it from deans and vice chancellors in the university exosystem? Or influences of the macrosystem which reflect a combination of learned academies, STEM culture in the macrosystem and history and life changing events in the chronosystem?

Gu and Day (2007) state that teacher’s resilience is relational, it is “determined by the interaction between the internal assets of the individual and the external environments in which the individual lives and grows (or does not grow)”, (p. 1314). Gu and Day (2007, 2013) also stress that resilience is rather less, innate, fixed and individual and more a multidimensional, multidetermined, dynamic and socially constructed concept. Thus, resilience varies from person to person and changes over time dependent on the circumstances, challenges and personal capacity to respond. Responses can be positive, with educational reform seen as an opportunity, and involving the individual asking: “what can I learn from this?”. Responses can also be negative and lead to hopelessness and depression (Mark & Smith, 2012). Academics’ development of resilience may depend on their sensitivity. Academics with higher “rejection sensitivity” will respond more negatively than others (Butler et al., 2007). Rejection sensitivity influences cognition, perception, self-regulation, emotion, motivation, and performance (Downey & Feldman, 1996; Kaiser & Kaplan, 2006; Pickett et al., 2004) and, due to the frequency of rejection in academia, the potential for developing rejection sensitivity is high (Day, 2011). Upon receiving a setback, ‘rejection sensitive’ academics may engage in higher social monitoring and scrutinise interactions with others to see if they will be rejected, try to manage others’ impressions of them by avoiding discussions of rejections, cognitively enhance the value of journals in which they have published (Pickett et al., 2004), or rely on dysfunctional coping mechanisms.

The measurement of quality for academics in education-focussed roles remains problematic. The shortcomings of metrics such as student evaluations and teaching awards which have been used to measure quality have recently received more negative attention than their benefits (Hamermesh & Parker, 2003; Sinclair & Kunda, 2000) with identified biases against females or culturally diverse non-native English speakers (Fan et al., 2019; Frederike et al., 2017; Kaschak, 1978; Sinclair & Kunda, 2000). These biases support current arguments that student evaluations should not be used for judging performance, tenure, and promotion (Zabaleta, 2007). Other metrics of quality such as student learning gain are difficult to measure, and it is problematic to identify as the effect of an individual academic when results are often part of a collective team effort.

In this study, responses of senior leaders in the exosystem illustrate the difficult work settings which impact the microsystem and the lives of STEM academics focussed on education. While some senior leaders were aware of the gap between rhetoric and reality and declared the value of STEM academics focussed on education and the adaptive capacity they gave to the institution, others questioned why there were STEM academics in education-focussed roles, thinking of them more as a misnomer, rather than a strategic intent. These two contrasting views, reflect the influence of the broader and more powerful exosystem. As a consequence, work settings have become for STEM academics focussed on education at best uncertain and at worse, antagonistic and insecure, and as a result stressful. In the last 2 years, the COVID-19 pandemic has intensified this stress with the rapid change to online learning, closures of international borders, academic redundancies and other job losses which have increased stress and caused a decline in academic staff health and well-being (Crawford et al., 2020; McGaughey et al., 2021; Mercado, 2020; Mok et al., 2020; Rapanta et al., 2020). Paradoxically the pandemic has created an environment where STEM academics focussed on education were never more needed and yet also at risk of losing their jobs.

Perceived lack of value of academics in education-focussed roles has potential negative flow on effects. Jordan (2013) describes that the consequences of being excluded from a cultural system is “experienced as urgent at a biological level as hunger, thirst or pain avoidance” (p. 72). A cultural system that denies the importance of connection …interferes with our ability to… to turn to others when in distress” (p. 74) and challenges the building of resilience. Jordan (2013) also describes a core motivation in life is connections. STEM academics in education-focussed roles described the lack of connections to communities, which were so naturally possible in a STEM academic role focussed on research. Mutual empathy and mutual empowerment, builds productivity and is a core motivation in life (Jordan, 2013). STEM academics focussed on education lack community and connections in their roles and need to build these, often found in the macrosystem rather than the micro and/or exosystem. This can be dangerous, because when there is a less powerful group, such as STEM academics in education-focussed positions, they are left to “make do” with rules of behaviour which are disempowering (Jordan, 2013, p. 77). As one interviewee graphically stated, if educational reform of the academic role creates two distinct communities, then “I think if you do that then you start to create a bit of a ghetto don’t you?” Interviewee 5, Level D, Go8.

Explanations for responses of senior leaders to STEM academics focussed on education may in part arise from the formal and informal influences in the macrosystem. Although the COVID-19 pandemic has intensified the stress on higher education (Watermeyer et al., 2021), increased competition linked to marketisation had made stress a ubiquitous feature of higher education prior to COVID-19 (Karlsen, 2013; Marginson, 2000, 2007; Norton et al., 2013). Across the globe, decades of assessments of research and education quality have been coupled with diminishing financial support from government to fund research and education. Increased student fees have also amplified expectations of students and parents about monetary return from higher education both during studying and afterwards, in careers (Deem, 2016; Deem et al., 2008; Marginson, 2000, 2007; Winter, 2009). Responses of senior leaders are also likely to arise from the disciplinary culture in science where there is a large gap between research and education. It is well known that science research success drives prestige, status, (Anderson et al., 2011; Savkar & Lokere, 2010), and these values govern academic work and identity (Becher & Trowler, 2001; Henkel, 2005, 2007). Bronfenbrenner’s model of relational resilience predicts that influences are reciprocal and bidirectional at the level of the microsystem. However, this does not appear to be the case when this model is applied to STEM academics in education-focussed roles. The microsystem is influenced in a unidirectional way from the macro to the exosystem and finally arrives in the microsystem. It could be that STEM research academics, especially the superstar researchers have capacity to influence the macrosystem because of their status and credibility created by research success.

STEM academics in education-focussed positions realised there was a challenge to upskill in education scholarship and research and build reputational expertise away from their disciplinary training. The gap in epistemologies and methodologies between disciplinary and education research is quite significant. Education research has a broader research paradigm, where scholars tend to commonly disagree (Gardner & Willey, 2016; Jones, 2011) and where methods may not use hypotheses formulation, e.g. grounded theory (Glaser, 1978). Depending on the discipline, STEM academics in education-focussed roles can find education scholarship and research confronting to understand, and time-consuming to learn (Simmons et al., 2013) and yet valuable when they have evidence of an effective teaching practice (NRC, 2012; Wieman, 2007; Wieman et al., 2010). Apart from the challenge of a different research paradigm, STEM academics found education research to be difficult for other reasons. First, they reported high teaching loads. Several academics commented that they could not complete education research because they were swamped by teaching and had no time. Second, there are fewer honours and postgraduate students or postdoctoral fellows who are interested in education research and fewer research assistants who have the skills to assist in it. Further, there was momentum required to create and run an education research group. Many interviewees commented on the difficulty and difference between the establishment of a disciplinary research lab compared to an education research group. They suggested that to promote engagement with education research required support for applying for education research grants, the formation and strengthening of intra-university education research, and the promotion of interdisciplinary education communities within universities where they can meet colleagues and explore potential research collaborations. Studies, by Trowler and Knight (2000) and Jawitz (2007), have shown that a community is critical to success because peers and their beliefs and values both support the socialisation process and decrease hindrances. Academics who responded proactively by creating or contributing to educational networks progressed. Academics who remained in the close confines of their schools or departments and were sensitive to negative views of education research as less than rigorous with low citations (i.e. “there is dross” in the education literature), or who were overwhelmed by the workload became increasingly isolated and stuck in a cycle of anxiety.

STEM academics were acutely aware of confusion in judgements and learned the hard way through failed promotion applications and also from the commentary by colleagues in the microsystem and deans in the exosystem. The longer STEM academics focussed on education are in higher education systems, and the more senior they become, the less likely they are to be mobile compared to their colleagues in STEM academic research roles. Although there are now more possible pathways for education-focussed academics inside universities for promotion there is a lack of positions advertised especially at senior levels. As one interviewee stated “No-one [who] comes through the door by stealth is a high-quality teaching and learning expert in science. That never ever happens”. Interviewee 7, Level E, ATN.

Academic identity and status is strongly linked to disciplinary research, cemented in PhD and postdoctoral training (Henkel, 2005). Transitioning into a STEM academic role focussed on education requires crossing an “identity threshold” (Simmons et al., 2013). In this study, some STEM academics optimistically pursued their new role to create opportunity and excitement to explore education theory and research (Flecknoe et al., 2017). Others reported doubt about the value of education research, mirroring the values of disciplinary colleagues and senior leadership. These academics appeared to be in a “liminal space” between disciplinary research and education practice and research, which was characterised by activity, but resulted not in crossing a threshold of understanding (Meyer & Land, 2005). A longer-term consequence of not crossing this threshold is that it can lead to ‘imposter syndrome’, a dual or chimeric identity which remains unresolved. Henkel (2005) suggests that individuals are both distinctive and socially embedded. However, “identities are, first and foremost, shaped and reinforced in and by strong and stable communities and the social processes generated within them” (p. 157). Being part of a community may override the discipline as being essential in forming a new academic identity.

How do STEM academics who are education focussed, build resilience and avoid what some of the interviewees in this study describe as a “handicap race”? Gu and Day (2007) argue that to build resilience in teachers requires a sense of belonging to a community and shared responsibility which enhances morale. Given resilience is relational and social, it can be enhanced or inhibited by social and environmental conditions (Gu & Day 2007, 2013; Gu, 2014). For STEM academics focussed on education, “communities of practice” have become important (Wegner, 1998). Communities of practice can be created at all levels of the system, at the microsystem level, by STEM education-focussed academics joining together within a specific discipline, at the level of the exosystem, where STEM academics focussed on education may come together at the faculty level and at the macrosystem level where organisations such as Councils of Deans or Academies may create conferences which provide support and influence culture to create a sense of belonging. These communities of people in the same situation who band together help each other form that identity (Trowler & Knight, 2000).

Resilient STEM academics in education-focussed roles were those who were able to adapt and learnt to successfully cross the threshold of research paradigms and navigate the journey from disciplinary to education research or scholarship. When faced with their educational or disciplinary-based research not being accepted by disciplinary researchers, senior leadership and promotion committees, they continued regardless. Other studies have found that deans view education roles as lower in status (Bush et al., 2020). STEM academics in education-focussed roles, who did not accept these views continued to progress even after failed promotion applications. Other academics who shared the same views, were not as resilient, and remained at the same level for several years. Rejection sensitivity could be part of an explanation for these responses. Day (2011) argues that the constant rejection can create “rejection sensitivity” which is a pivotal driving force in stifling productivity, creativity and innovation and therefore shaping future academic identities. Rejection in peer review or grant applications is not only an academic rejection of ideas, but also a rejection of membership to the social circle of successful scholars (Day, 2011). Such is the power of rejection, that it can lead to social isolation, reduced effort, avoidance of research, and ultimately forces some to leave academia (Day, 2011). There have been suggestions, that solutions to this are a more fluid model of the academic workforce. Coates & Goedegeburre (2012) described a model where academics could transition between research and education at different stages of their careers. Even for the most resilient academics, models of the academic workforce as suggested by Coates & Goedegeburre (2012) were not observed in this study. No academics interviewed had moved from STEM disciplinary research to STEM education-focussed research or disciplinary-based education research and then back to disciplinary research. In this current climate of super competitiveness (Carson et al., 2013), this workforce model in a science culture seems an ideal, but also a delusion.

In conclusion, STEM academics in education-focussed roles face a troubled future, understanding the individual and influences and interactions between the micro, exo and macrosystems will help to build resilience. During this pressured decade, where COVID-19 has intensified stress, more attention is needed on the relationships in the socio-ecological model in order for educational reform of the academic role in higher education STEM to be effective.

## Data Availability

The data sets generated and analysed during the current study are not publicly available due to the identifiable nature of the data.
